# In Situ Characterization of Micro-Vibration in Natural Latex Membrane Resembling Tympanic Membrane Functionally Using Optical Doppler Tomography

**DOI:** 10.3390/s20010064

**Published:** 2019-12-20

**Authors:** Daewoon Seong, Jaehwan Kwon, Deokmin Jeon, Ruchire Eranga Wijesinghe, Jaeyul Lee, Naresh Kumar Ravichandran, Sangyeob Han, Junsoo Lee, Pilun Kim, Mansik Jeon, Jeehyun Kim

**Affiliations:** 1 School of Electronics Engineering, College of IT engineering, Kyungpook National University, Daegu 41566, Korea; smc7095@knu.ac.kr (D.S.); luffy6462@naver.com (J.K.); dmjeon@knu.ac.kr (D.J.); jaeyul@knu.ac.kr (J.L.); nareshr.9169@gmail.com (N.K.R.); progkiller@paran.com (S.H.); jslee5399@knu.ac.kr (J.L.);; 2 Department of Biomedical Engineering, College of Engineering, Kyungil University, Gyeongsan 38428, Korea; eranga@kiu.kr; 3 School of Medicine, Institute of Biomedical Engineering, Kyungpook National University, Daegu 41944, Korea; pukim@knu.ac.kr

**Keywords:** micro-vibration, latex membrane, localized distribution of sound, optical Doppler tomography

## Abstract

Non-invasive characterization of micro-vibrations in the tympanic membrane (TM) excited by external sound waves is considered as a promising and essential diagnosis in modern otolaryngology. To verify the possibility of measuring and discriminating the vibrating pattern of TM, here we describe a micro-vibration measurement method of latex membrane resembling the TM. The measurements are obtained with an externally generated audio stimuli of 2.0, 2.2, 2.8, 3.1 and 3.2 kHz, and their respective vibrations based tomographic, volumetric and quantitative evaluations were acquired using optical Doppler tomography (ODT). The micro oscillations and structural changes which occurred due to diverse frequencies are measured with sufficient accuracy using a highly sensitive ODT system implied phase subtraction method. The obtained results demonstrated the capability of measuring and analyzing the complex varying micro-vibration of the membrane according to implied sound frequency.

## 1. Introduction

The human tympanic membrane (TM) is one of the major parts of the middle ear that transmits audible sound waves to the cochlea [1,2]. Diseases, such as bullous myringitis and traumatic TM perforation cause structural changes and malfunctions, which alter the vibration patterns, obstructing hearing and communication. In addition, hearing loss, middle ear effusion and otosclerosis among other tympanic diseases induce a change in the vibration pattern of the middle ear, eventually leading to hearing disorders [3,4]. Among many ear-related diseases, hearing loss is a worldwide pervasive disease, and can be broadly classified into noise-induced hearing loss caused by ambient noise and age-related hearing loss [5,6]. 

The conductive hearing loss is occurred in the outer or middle ear, where sound waves are not able to carry all the way through to the inner ear. Audio signals can be blocked by earwax or a foreign object located in the ear canal, and therefore, the middle ear space may be impacted with fluid, infection or a bone abnormality along with eardrum injuries. Conductive hearing loss is mostly common in children with recurrent ear infections or foreign objects in their ear canal [7]. Sensorineural hearing loss is another type of hearing loss, which occurs due to damages of the inner ear or the actual hearing nerve. This loss generally occurs when some of the hair cells within the cochlea are damaged. Sensorineural loss is the most common type of hearing loss, and it can be a result of aging, exposure to loud noise and injuries. This type of hearing loss is typically not medically nor surgically treatable [8]. The next abnormality is the combination of both sensorineural and conductive hearing loss. They may have a sensorineural hearing loss and then develop a conductive component in addition. Therefore, a pre-identification method to diagnose ear-related diseases by measuring the micro-vibrations of the TM can be considered as a valuable technique [9] for precise diagnosis. However, due to the aforementioned physiological nature of the diseases, most of the developed audiological instruments have been potentially employed to examine conductive hearing compared to sensorineural loss and mixed hearing loss.

The most common approach has been, and is still largely in use as the gold standard, pure tone audiometry, which has been used for more than 50 years to diagnose and evaluate the degree of hearing loss in present otolaryngology [10]. However, despite the results obtained in this inspection method has been based on the subjective reaction of the patient by stimulation and cannot be used for infants [11]. Moreover, the lack of appropriate treatment and difficulty of accurate pre-diagnosis have caused many young people to suffer from noise-induced hearing loss due to excessive use of earphones [12].

The aforementioned limitations have long been known, and in response, alternative noninvasive investigations have gained significant attention. Hence to diagnose ear-related diseases caused by abnormal vibration patterns in TM, laser Doppler vibrometry (LDV) and stroboscopic holography were developed and have been actively studied [13,14]. LDV was fundamentally developed as a non-contact diagnostic tool to measure the vibration of a surface. When the LDV is directed at the TM surface, and the amplitude and frequency of the vibrations are extracted from the Doppler shift (occurring due to TM surface motion) of the reflected laser beam frequency [13]. Fast single point detection of this technique allows general diagnosis, and enables an identification of ossicular chain pathologies of living subjects [15,16]. However, the difficulty of providing depth-resolved information and large time consuming for whole range of TM inspection limit the applications of the technique [17,18]. Stroboscopic holography is one of the recently presented methods which measure the amplitude and phase of TM displacement [19,20]. Although this method allows us to observe the vibrating motion of the entire TM as well as three-dimensional (3D) surface motion [21,22], the insufficient movement data of the morphological internal structure and prolonged time for measurements due to frequency resolution limits the extensive applicability [23]. Thus, afore-stated, non-invasive methods either are only sensitive to surface properties, or do not have sufficient axial and spatial resolution to be useful for the overall measurement and analysis of structural thickness. 

To overcome afore-stated drawbacks, a non-invasive and high-resolution, low-coherence interferometric imaging technique called optical coherence tomography (OCT) was involved to obtain depth-resolved images in real time [24,25,26]. OCT is used in various fields, such as dentistry [27,28], agriculture [29,30] and even entomology [31,32]. OCT has been actively used in various medical fields, including diverse otolaryngological studies, which detect the pathological changes of TM [33,34], and to study the anatomical structures and for measuring thickness of TM in volumetric images [35]. Although OCT is capable of detecting real-time structural movements, distinguishing single-pixel difference-based micro-vibrations and fine movements are limited. Optical Doppler tomography (ODT) is an extension of OCT, which was initially developed to measure fluid flow [36,37]. ODT adds an algorithm to the detect movement information of a sample by measuring the phase shift of the detected interference signal. 

ODT-based experiments can be performed simply by adding a software algorithm without installing additional hardware to an OCT system. Numerous algorithms, such as intensity-based Doppler variance, phase-resolved Doppler variance and phase-resolved color Doppler assessments using phase difference, were developed and widely used to detect the Doppler signal [38,39]. Moreover, the ODT technique has been used for angiography, as it can measure the blood flow while imaging the monolayer structure [40,41]. Furthermore, ODT algorithms were developed and actively studied for skin tissues, ophthalmic angiography [42] and human TM [43,44,45,46]. Middle ear anatomy and functions were investigated using phase-sensitive, OCT-integrated video otoscopy in a previous attempt. However, video otoscopy has a limitation of examining micro-vibrations of the middle ear, and the post-processed phase information was obtained with a limited imaging range [47]. OCT-vibrography and OCT-integrated, surgical, microscopy-based imaging techniques were demonstrated at numerous attempts to map the phase information of the ossicular chain motion and diagnose middle functionality, where the conclusions were made based on the post-processed data assessments with limited real-time assessments [48,49,50].

In this demonstration, we measured phase shift values using ODT to assess micro-vibrations of a natural latex membrane, which can be adapted as a representative sample of preliminary study for measuring vibration patterns in the human TM. In comparison with existing methods, the audio signal frequency and the sampling rate were matched accordingly to examine particular vibration patterns and circular mode vibrations-based Doppler signals and phase information of the latex sample, which resembles TM vibrations. To demonstrate the feasibility of measuring micro-vibration using the ODT technique, we extracted the micro-oscillations of the sample surface from two dimensional (2D) and three dimensional (3D) ODT images, illustrating the overall motion of the entire latex sample. In addition, a graphical analysis was conducted on the acquired images to quantitatively analyze the vibrating motions of the sample structure. 

## 2. Materials and Method

### 2.1. Experimental Specimen Preparation

The micro-vibrations of a natural latex sample induced by two speakers were acquired and processed to evaluate the capabilities of the developed system, in order to detect the complex vibration patterns in a sample structure. The measured thickness of the latex membrane is 80 µm. The membrane is placed on a 5 mm diameter metal tube, which is pulled tight through the translation stage. We used a set of two speakers to make a localized distribution of sound on the latex membrane, resembling the biological response of the TM to compensate for the equivalent anatomical characteristics. The output sound of the speakers was connected to a computer (PC), controlled by software using a beep function, which can change the frequency of the sound. Since the utilized frequency band is sufficient to measure the micro-vibration of the latex membrane and verify the capability of this ODT technique, frequencies lower than 2 kHz, and especially under 1 kHz, were not used in this study. To barricade external sound drawbacks, the galvanometer scanner, the latex membrane (sample) and audio devices were installed in a customized soundproof chamber. The detected sound pressure in the chamber is 12.4 dB lower than the 39.2 dB which is the external sound pressure.

**Figure 1:**
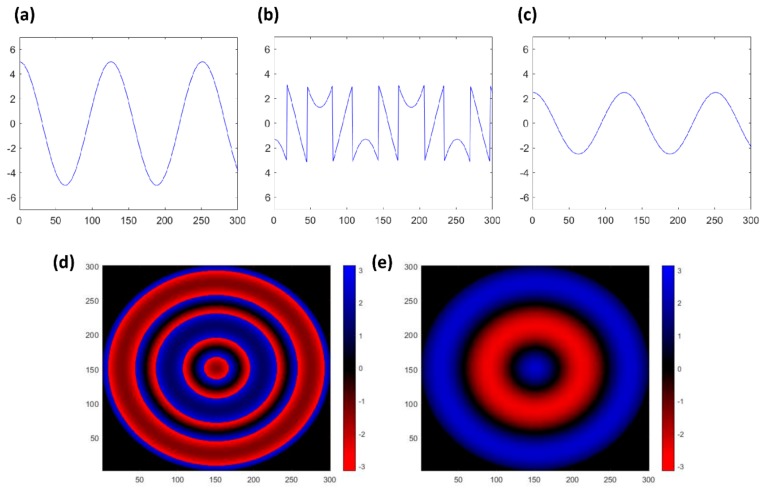
(**a**) Single cosine wave, which amplitude exceeds the intended range. (**b**) Wrapping phase graph of (**a**). (**c**) Amplitude-controlled cosine waveform. (**d**) 2D phase mapping image of (**b**). (**e**) 2D phase mapping image of (**c**).

The utilized frequency was selected from 2 to 5 kHz, which is within the audiometry frequency range [51] to verify capability of the ODT technique for detecting fine vibration. Figure 1 represents the reason of controlling the applied sound pressure to the latex membrane. Figure 1a is a single cosine waveform, and its amplitude exceeds the intended range of −π to + π, which is our set phase range in this experiment. Figure 1b is the wrapped waveform of Figure 1a due to the selected phase range. In contrast, Figure 1c shows the amplitude-regulated cosine wave of Figure 1a to be matched in the intended phase range, which is the same process of regulating the sound pressure of the speaker in our experiment. By utilizing the waveform of Figure 1b,c, we obtained the phase mapping image shown in Figure 1d,e, respectively. The color map is equivalent in Figure 1d,e. 

The plotted signal image of Figure 1d becomes more complex compared to Figure 1e due to wave folding of the cosine wave shown in Figure 1b caused by the limited amplitude. In addition, even with the same phase value, it is difficult to determine whether the obtained result is original vibrating data or wrapped data due to the bi-directional vibration characteristic. The simulated case using a single cosine wave described in Figure 1 shows a significantly different result caused by wrapping. Since measuring the vibration of the latex membrane is even more complex than in the single wave case, we controlled the sound pressure to limit the phase value within an intended range and avoid the case of applying an unwrapping method according to the purpose of our paper.

### 2.2. Description of the ODT System Configurations

Figure 1a depicts the schematic of the ODT system, which is used in the present study assembled with a sound chamber consisting of a set of speakers. The broadband light source of the low-coherence interferometer was a super-luminescent diode (EXS210022-02, Exalos, Swiss) with a center wavelength of 840 nm and full width at half maximum (FWHM) of 50 nm. The laser beam output from the light source was passed into the optical fiber coupler (50:50, TW850R5A2, Thorlabs, New Jersey, USA). The light was divided evenly into a sample arm and a reference arm thorough the optical fiber coupler. The 3D scanning is operated by using a galvanometer scanner (GVS002, Thorlabs, NJ, USA), which moved the light along the x and y axes. At the sample arm, light was scanned over the entire latex membrane, covering 600 × 600 (x × y) spatial points. To produce the micro-vibration in the latex membrane, two speakers are placed at opposite lateral sides to the latex. The two backscattered light beams from the reference arm and sample arms were interfered, and the generated interference signals were transmitted to the optical spectrometer, which contains a collimator (F810APC-842, Thorlabs, NJ, USA), a broadband dielectric mirror (BB2-E03, Thorlabs, USA), an achromatic doublet (AC508-100-B, Thorlabs, NJ, USA), diffraction grating (spatial frequency 1800 lpmm, Wasatch Photonics, USA), which is used to diffract signals along each wavelength and a 4096-pixel line scan camera (spL4096-140 km, Basler, Germany). 

The lateral resolution was measured as 15.6 μm using a resolution target (USAF 1951, Edmund Optics, USA) and the theoretically calculated axial resolution was 6.23 μm (in air). In addition, the measured SNR using the multiply acquired mirror images-based maximum point spread function (PSF) signals of the built system was 30.9 dB. SNR was used as a quantitative metric to evaluate the improvement in the images due to the speckle reduction. SNR measures the strength of an object in the image in the presence of background noise. The SNR measurement can be expressed as,

$$SNR=10\log_{10}(\max[\frac{B_{lin}^{2}}{\sigma_{lin}^{2}}])$$

where the numerator represents the linear magnitude of the optical signal and the denominator represents the linear magnitude of the background noise signal.

**Figure 2:**
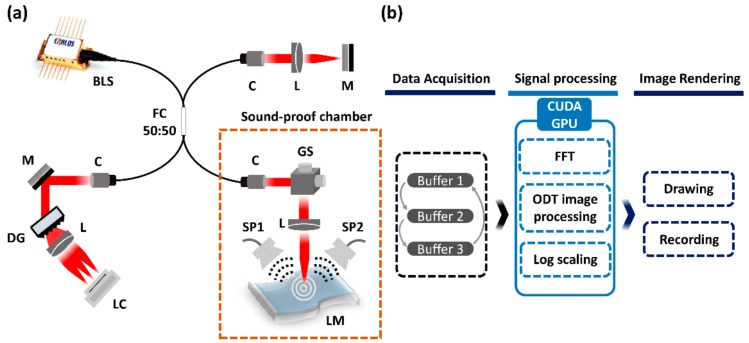
(**a**) Schematic of the Optical Doppler tomography (ODT) system. BLS, broadband laser source; C, collimator; DG, diffraction grating; FC, fiber coupler; GS, Galvanometer scanner; L, lens; LC, line scanning camera; LM, latex membrane; M, mirror; SP, speaker. (**b**) Software algorithm of the parallel signal processing for the Doppler experiment.

The flow chart of the microvibration measurement procedure is shown in Figure 2b. The software configuration is divided into data acquisition, signal processing and plotting. Each category is operated by multithread technique using the ring buffer-enabling signal acquisition, signal processing and visualization simultaneously [52]. The algorithm developed for the high-speed parallel processing, using a compute unified device architecture (CUDA) (Nvidia, Santa Clara, CA, US) with a graphics processing units (GPU) programming tool, was applied to the signal processing section which handles most of the algorithm part.

### 2.3. Principles of ODT Imaging and Processing

To obtain consistent results, sound wave generating speakers and the galvanometer scanners were synchronized simultaneously, since the non-synchronization between the afore parameters and frequency obstruct the acquisition of the exact scanning position, which comes across with the particular frequency. Thus, the time synchronization between A-line period and frequency time period insures our measuring generated entire membrane vibrations, especially the specific vibrating mode of the latex membrane, with consistent amplitude and phase of applied sound waveform. In the current demonstration, the fundamental scope was to verify specific circular mode vibrations of the latex membrane in accordance with various sound frequencies. Although more vibration information can be acquired according to the Nyquist sampling law by employing a sampling rate that is twice higher than the frequency, sound waves with similar sampling rates and frequencies were applied to each point of the sample with a different A-line frequency. 

Since the acquired sinusoidal vibration patterns sufficiently confirmed the circular mode vibration patterns of the latex sample according to the audio frequencies, Nyquist sampling was not implemented in the current assessment. The total detected time period is 500 μs for the initial 2 kHz frequency, as shown in Table 1. The regulated A-line time was varied from 455 to 313 μs according to the applied frequency. The dimensions of a single C-scan (600 × 600 × 600 pixels) and the time consumed for the image acquisition and the total taken time for scanning 5 mm × 5 mm were 180, 163.8, 128.5, 116.3 and 112.7 s at 2, 2.2, 2.8, 3.1 and 3.2 kHz, respectively. This was attained by setting the scanning speed of the ODT system in accordance to the applied frequencies.

The acquired raw data was Fourier transformed and converted to a digital signal to obtain the amplitude and phase information of the detected optical interference signal. The acquired amplitude was plotted in logarithmical scale, and phase information was used in the Kasai autocorrelation equation to derive the Doppler frequency shift [37]. The arctangent function evaluates the average phase shift between each obtained phase data in order, and was computed in all four quadrants, depending on the degree of vibration [53,54]. The 2-sided vibrating directions were represented with opposite signs from − π to + π. As the magnitude of the vibration was increased, the absolute value of the analyzed phase shift was getting closer to + π. After calculating to obtain Doppler phase shift, color mapping of the ODT images was determined according to the phase shift values. Furthermore, an additional noise was produced by the galvanometer scanners, which directly affect the vibrating pattern of the latex membrane. Thus, to minimize the noise effect, the phase subtraction method, which is an additional software processing method, was developed to minimize the external noise. The mechanism of the phase subtraction method is employed to identify the detected phase values at stationary state from the measured values at the stimulated state. Every phase data when measured before applying sound was saved and used to extract the noise.

## 3. Results 

### 3.1. Analysis of the Reasons for Using Dual Speakers

**Figure 3:**
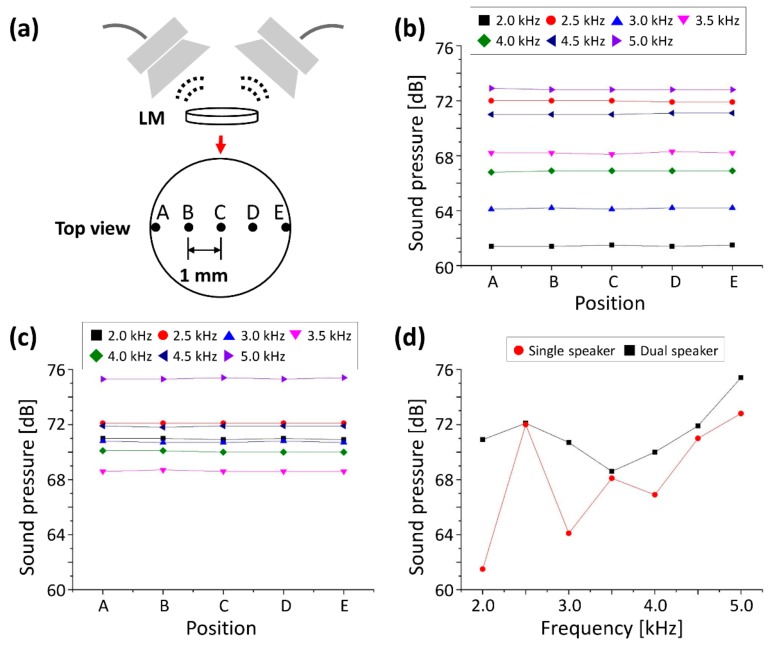
The quantified sound pressure graphs of applied sound using a single and dual speaker. (**a**) Detected positions of sound pressure in the latex membrane (A–E). (**b**) Detected sound pressures within the frequency range of 2 kHz to 5 kHz using single speaker configuration. (**c**) Detected sound pressures with equivalent condition of (**b**) using dual speaker configuration. (**d**) Comparative graphical analysis of the obtained results using single speaker and dual speaker configuration.

To verify the validity of using a dual speaker unit, the results of sound pressure detected using a single speaker and dual speaker were compared in Figure 3. Figure 3a demonstrates the detecting points of sound pressure in the latex membrane from A to E, separated by a distance of 1 mm from each other. The sound pressure measurements of each individual location (from A ~ E) on the latex membrane, while using single and dual speaker configurations, were measured using a commercial decibel meter (Model: UT-353, M-TECH, Korea). Figure 3b shows the detected pressure generated by a single speaker in the respective positions from A–E. Similarly, we measured the acoustic pressure on the sample generated by dual speaker configuration, which is shown in Figure 3c. Figure 3b,c depict the detected sound pressure at A–E for the generated, various sound frequencies from 2 kHz to 5 kHz. It was confirmed that the tendency of sound pressure at each frequency was different depending on the speaker configuration. The sound pressure was adjusted to 70 dB to create a similar condition with pure tone audiometry used in the field [55,56]. To visually compare the sound pressure tendency at a glance, the detected sound pressures at point C are shown in Figure 3d using detected values of Figure 3b,c. Compared with the results using the single speaker, the detected sound pressure at each position using a dual speaker was different. Furthermore, we confirmed that the applied audio signal was complex as well, and formed different vibratory motion when compared to the vibration pattern generated using a single speaker.

The dual speaker configuration was adopted to form a more complex vibratory pattern from the applied audio signals. Since the result revealed that the detected sound pressure at each point was identical when we applied constant frequency in both configurations, while the value of sound pressure was varied according to modulation of the frequency, it was confirmed that the applied audio signals using a dual speaker can make a more complex motion of the latex membrane, which can demonstrate maximum capabilities of the developed ODT system for detecting the micro-vibration the of latex membrane at more complex audio signals.

### 3.2. OCT and ODT Cross-Sectional Assessments for Micro-Vibrations

**Figure 4:**
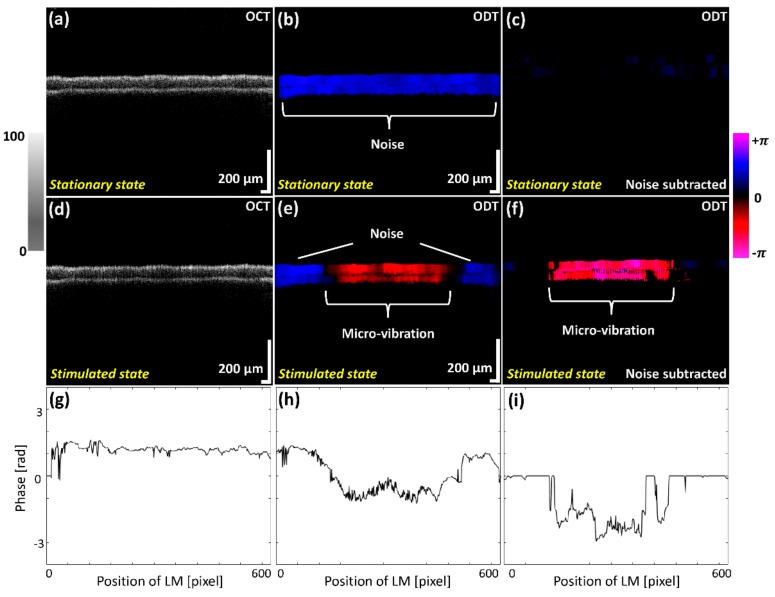
Two dimensional optical coherence tomography (2D-OCT) and Optical Doppler tomography (ODT) images acquired before and after exposing the latex membrane to the sound waves. (**a**) 2D-OCT image of latex membrane before applying sound. (**d**) 2D-OCT image of vibrating latex membrane. After Doppler image processing, (**b**,**e**) are 2D-ODT images with severe noise. (**c**,**f**) are processed 2D-ODT images with an algorithm to subtract phase differences before and after sound is applied, eliminating unnecessary noise at (**b**,**e**). (**g**–**i**) are indicated the phase data of (**b**), (**e**) and (**f**), respectively.

The micro-vibrations were investigated using OCT and ODT cross-sectional images while scanning the membrane before and after exposing to the diverse frequency ranges. Figure 4 shows the OCT and ODT results of the two different states before applying sound indicated as a stationary state, and after applying sound expressed as a stimulated state. The illustrated OCT and ODT results of Figure 4a,b were obtained during the absence of sound waves (control), where Figure 4d,e represent oscillation images of the latex membrane, which were acquired while providing a 2 kHz sound wave frequency. Although vibration effects can be slightly recognized in the grayscale OCT image of Figure 4d, identification of prominent fine oscillations is difficult to distinguish. Slightly occurred phase shift evaluations caused by micro-vibrations with external noise effect were color mapped in ODT results, as shown in Figure 4b,e. 

In order to eliminate the aforementioned noise effect, we used the phase subtraction method, comparing the phase difference before and after applying sound. The results of Figure 4c,f demonstrate the ODT images with the applied noise filtering software to minimize the unnecessary noise effect of Figure 4b,e. Although erroneous numerical values have been significantly reduced comparatively, slight noise signals were still left and unable to be removed in the (+) direction due to the sensitivity of measuring micro-vibrations and the inconsistency of phase shift generated by scanners. To quantitatively validate the effect of the phase subtraction method on the ODT image, the phase data of Figure 4b, e and f were plotted in Figure 4g–i. Figure 4g shows the initial noise generated from the external aforementioned factors. In addition, the result of removing a redundant noise, which is shown in Figure 4g, was verified by comparing with Figure 4h,i. After subtracting the noise, the pure fine vibration of the latex membrane was measured in this study. These results revealed that the noise removal was sufficiently accomplished to verify the micro-vibration of whole latex membrane.

### 3.3. Enface-ODT Representation with Distinct Frequencies

**Figure 5:**
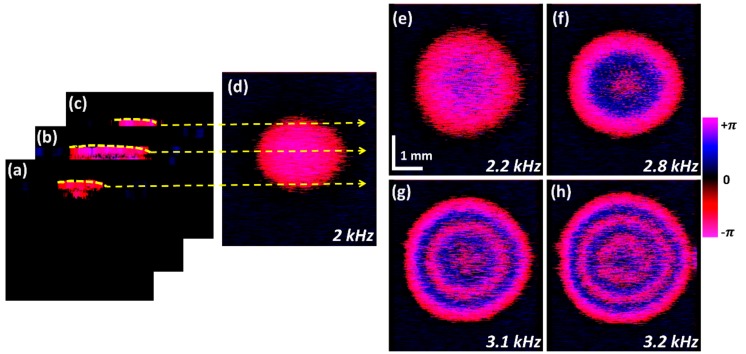
Process to obtain 2D-ODT enface image representations. (**a**–**d**) Process of 2D-ODT enface image extraction. (**a**–**c**) depict acquisition of uppermost cross-sectional information. (**d**) Total ODT-enface representation at 2 kHz frequency. (**e**–**h**) show the ODT enface image variations according at 2.2 kHz, 2.8 kHz, 3.1 kHz and 3.2 kHz frequencies, respectively.

To identify the overall oscillation pattern of the entire latex membrane and circular mode of vibration, we extracted enface information from the uppermost pixel layer of each cross-sectional image. Figure 5a–c represent the aforementioned ODT enface image construction process for representative three images among 600 points of the y axis using the open source computer vision (OpenCV 2.3.1) image processing tool served by Intel. Figure 5d emphasizes the ODT-enface representation of the natural latex membrane micro-vibrations at 2 kHz, and the measured sound pressure was 70.9 dB. 

The result reveals that the pattern of the vibration of the latex membrane is formed throughout the sample surface. This shows the capabilities of the proposed system to assess the micro-vibrations on the sample structure acquired using the developed ODT system. In Figure 5, we also used the phase subtraction method to remove external noise during C-scan imaging. The C-scan images were acquired using 600 B-scan images (with phase information), and the noise of each B-scan image was different from another. Hence, we first scanned the range of the x and y axes before exposing the membrane to the sound and saved all the measured values. Later, the saved noise values were subtracted from each B-scan.

To validate the obtained results, we conducted additional experiments with different frequencies. Figure 5e depicts the vibrating ODT enface image at 2.2 kHz, and in comparison with Figure 5d, the degree of phase shift is observed to be increasing towards the positive direction. Figure 5f–h show processed ODT enface images at 2.8 kHz, 3.1 kHz and 3.2 kHz, respectively. Each frequency of Figure 5d–h was selected when the ODT image was significantly different compared with other results. The sound pressures detected on the latex membrane at each frequency were 70.9, 72.7, 67.4, 71.4 and 70.3 dB, which is shown as the black square in Figure 3d. Obtained vibration patterns according to varying frequencies were differentiated as vibratory modes from (0, 0) to (0, 6). Where the vibratory mode (0, 0) is the vibratory pattern observed in the sample structure with the least phase value of vibratory. This can be seen in Figure 5d, where the phase of the sample structure is more or less even throughout the sample surface. The vibratory mode (0, 6) is the maximum observed phase value of vibratory motion of the sample structure. This is shown in Figure 5h, here the varying phase from − π to + π can be seen as multiple concentric circular rings with varying intensity from magenta to blue. The detected various circular modes of vibration confirm the applicability of an ODT system to measure the micro-vibrations in a sample, which is subjective to varying frequencies. The 3D graphs plotted by using equivalent phase data are shown in Figure S1 to demonstrate the entire vibrating motion visually at a glance.

### 3.4. Phase Shift Assessment of Latex Membrane Micro-Vibrations

**Figure 6:**
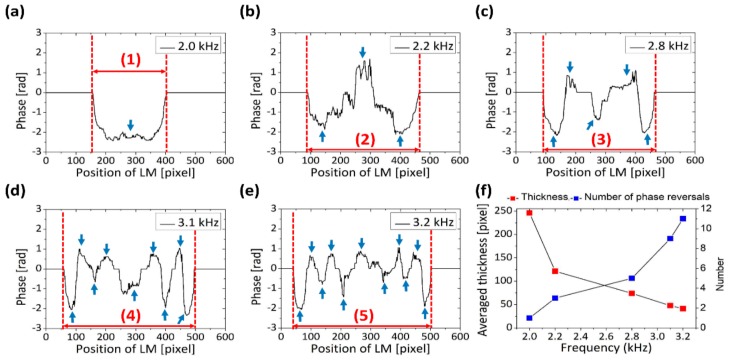
Graphical analysis based on data of 2D ODT surface images. Images (**a**–**e**) are using averaged raw phase data for scanning position and its respective vibrations obtained by varying frequency at 2, 2.2, 2.8, 3.1 and 3.2 kHz. The x axis represents the position of latex membrane, and the y axis indicates phase information from − 3 to +3 in all five graphs. Blue arrows at (**a**–**e**) show the points of counting phase reversal. Graph (**f**) depicts the number of phase reversals and averaged vibrating distance for each frequency, and (**f**) is plotted using the obtained data of (**a**–**e**). LM, latex membrane.

The obtained ODT-enface representations of Section 3.2 underwent on phase shift assessment as shown in Figure 6. The graphs were plotted by employing the phase information measured at each point in the latex membrane using MATLAB software and Origin Pro to show the results quantitatively and more visually. 

In order to minimize the effects of noise, and to verify the overall vibrational pattern of the latex membrane, we averaged 10 consecutive images acquired from the center region. The averaged phase information was filtered using a median filter to plot the graphs, which are shown in Figure 6a–e. Each graph is plotted with phase information at scanning points around the center point while applying respective frequencies, such as 2 kHz, 2.2 kHz, 2.8 kHz, 3.1 kHz and 3.2 kHz. Figure 6a shows that the entire latex membrane moves towards a single direction, whereas Figure 6b shows that the number of oscillations of this membrane increases compared to Figure 5a. Moreover, accumulative direction changes of vibrations were clearly identified along with the frequency increase as shown in Figure 6c–e.

According to the demonstrated quantifications, it was revealed that the number of oscillations is proportional to the applied frequency. In order to quantitatively analyze the micro-vibration, we measured the averaged thickness value of the concentric circular rings in Figure 5d–h, and this is plotted and shown in Figure 6a–e. The number of phase reversals in Figure 6a–e were counted when the sign of phase was different compared with the previous value. The points of phase reversals are indicated with a blue arrow in Figure 6a–e, and total phase reversals are 1, 3, 5, 9 and 11, which increased with respect to the applied frequency. In addition, these total number of measured phase reversals are plotted with blue square points in Figure 6f, and the red square points represents the measured averaged thickness. The values used to measure the total vibrating range of the latex membrane according to the applied frequency are indicated numerically from (1)–(5) in Figure 6. We divided each vibrating portion into the aforementioned number of phase reversals to verify averaged thickness of oscillation. Since the entire latex membrane vibrates in a single direction (vertical) at 2 kHz, the value is set to 243 pixels. The total averaged displacement of the latex membrane for the given frequencies of 2.2 kHz, 2.8 kHz, 3.1 kHz and 3.2 kHz were measured as 124, 76, 48 and 44 pixels, respectively as indicated in Figure 6f with red color square points. The acquired vibrating distances exhibited a decreasing tendency, confirming the complexity of vibrating motion with the increase of frequency. Thus, we verified that the results of our experiments are consistent, and the measurement of the frequency-dependent micro-vibrations is possible with the proposed method. The process involved in plotting Figure 6 is explained in Figure S2.

## 4. Discussion

According to quantitatively analyzed results aforementioned in the manuscript, we have demonstrated the possibility of detecting the micro-vibration and complex vibratory pattern using our proposed system. In order to improve the efficiency scanning time in the developed systems for measuring the complex vibratory patterns, a fast scanning speed was achieved by adopting CUDA multi-thread processing. The total time taken to image a sample with a scan range of 5 mm × 5 mm was 180 s with an applied frequency of 2 kHz (as the audio signal applied to the sample). Similarly, the total time taken for measuring the micro-vibration in sample for frequencies 2.2, 2.8, 3.1, 3.2 kHz was 163.8, 128.5, 116.3 and 112.7 s, respectively. The total time taken for scanning the sample using our ODT system is much faster when compared to LDV and stroboscopic holography for an equivalent scan region [18,23]. In addition, ODT also offers a depth-resolved, morphological structure of the stimulated sample. The utilized frequency band in this study is slightly different as those used in previous audiometry result [57]. However, the utilized sound frequency was selected according to the audiometry frequency range [51], and this range was sufficient to induce fine vibration patterns of the latex membrane. In addition, the A-scan time is adjustable even more than 10 kHz to cover a broad range, which is yet to be reported.

In this study, we used a natural latex membrane to approximate the condition with the human TM for detecting micro-vibrations on a sample surface [58,59]. However, due to differences of natural parameters like thickness and elasticity between the latex membrane and the TM, the limitations about creating a complex vibratory motion on the latex were remained. To overcome this problem, we generated the localized sound distribution by using a dual speaker configuration to create a more complex vibratory motion on the latex surface similar to the TM. We measured the complex vibratory motion and observed the respective vibratory patterns on the sample surface for frequency ranges within the human audible range. Compared to the previously reported single speaker configuration-based stroboscopic holography [23], multiple concentric circular ring patterns were induced on the sample by employing a dual speaker configuration. The results revealed the possibility of achieving more complex vibratory motions of sample structure and the occurrence of a concentric circular pattern increased along with increase of the frequency level.

In addition, we focused to measure the changing rate of phase value in each position of the latex membrane at varying frequencies. Amplitude can be interpreted as other parameters, which include physical vibratory motion and intensity differences in the OCT image. However, as the physical thickness in the utilized natural latex is consistently even across the entire sample, we considered that the magnitude of vibration is not significantly different for the entire sample. Furthermore, layer intensities of the OCT image are not sufficient for obtaining the micro-vibration pattern of the latex membrane. In case of the varying phase values, it changes in accordance to the vibratory motion of the sample structure. For these reasons, we measured the rate of change in phase value in each position of the latex membrane at varying frequencies.

In the current study, phase data unwrapping was not performed, since phase unwrapping is commonly utilized in Doppler experiments, which focuses on measuring the velocity of a liquid flow within vessels or in capillary tubes. In case of the velocity measurement of fluid flow, the path of the transmitted fluid is usually in one particular direction (either forward or backward), hence phase unwrapping can be effectively used to analyze the total phase shift occurring in the detected signals. However, the primary scope of the study was to determine the vibration pattern in this latex membrane, which represents the oscillations of the (natural) tympanic membrane. We maintained the experimental conditions, where the vibrating surface does not exceed the intended range [55,56] of phase shift (i.e., − π to + π). This was achieved by maintaining the desired audio signal amplitude of the speakers to regulate the sound pressure applied to the sample. This in turn excluded the necessity to implement the phase unwrapping, which is commonly used to measure the phase changes that exceed the range of −π to + π.

## 5. Conclusions

In this study, the two-dimensional (2D) and three-dimensional (3D) ODT evaluations were well-utilized to assess the micro-vibrations of natural latex membranes for diverse frequencies resembling the structural displacement of human TM. Compared to existing methods, such as LDV and stroboscopic holography, the ODT technique is substantially capable of measuring micro-vibration, and consequently providing the depth information of the entire latex membrane with a short scanning time. The entire motion of the latex membrane was analyzed through cross-sections, and the phase shift was analyzed through the 3D volumetric assessments. The externally influenced noise subtraction was additionally included in the ODT algorithm to enhance the precise classification. The acquired results from the localized distribution of sound confirmed that the vibrating direction, as well as the complex oscillations, were proportionally increased along with the gradually rising frequency. Compared to the existing methods, the sampling rate was matched with the applied sound frequency to verify particular vibrating patterns. Furthermore, an escalation in the order of circular mode was identified along with higher frequency levels. Subsequently, the proposed ODT inspection method reveals the possibility of using it as a measuring method for the different complex micro-vibration of the membrane depending on the applied sound frequency. As a further extension of the work, the acquired results of this study will be used as baseline threshold parameters for future human TM assessments to confirm the detection capability of multiple frequency-based micro-vibrations using the ODT methodology.

## Figures and Tables

**Table 1:** Measured scan range in this experiment, A-line time and total time taken for scanning at 2, 2.2, 2.8, 3.1 and 3.2 kHz.

Scan Range = 5 mm × 5 mm					
Frequency (kHz)	2	2.2	2.8	3.1	3.2
A-line time (μsec)	500	455	357	323	313
Total time (sec)	180	163.8	128.5	116.3	112.7

## Supplementary Materials

The following are available online at https://www.mdpi.com/1424-8220/20/1/64/s1, Figure S1: 3D-ODT mapping information to visualize direction of oscillation and correlated phase information for each scanning point, Figure S2: Entire process of plotting the Figure 5 using an example of 2D-ODT examples.

## References

[1] Volandri G., Di Puccio F., Forte P., Carmignani C. (2011). Biomechanics of the tympanic membrane. J. Biomech..

[2] Tonndorf J., Khanna S.M. (1970). The role of the tympanic membrane in middle ear transmission. Ann. Otol. Rhinol. Laryngol..

[3] Majima Y., Takeuchi K., Hamaguchi Y., Morishita A., Hirata K., Sakakura Y. (1988). Hearing impairment in relation to viscoelasticity of middle ear effusions in children. Ann. Otol. Rhinol. Laryngol..

[4] Chole R.A., McKenna M. (2001). Pathophysiology of otosclerosis. Otol. Neurotol..

[5] Lin F.R., Niparko J.K., Ferrucci L. (2011). Hearing loss prevalence in the United States. Arch. Intern. Med..

[6] Liu X., Yan D. (2007). Ageing and hearing loss. J. Pathol..

[7] Moore D.R., Hine J.E., Jiang Z.D., Matsuda H., Parsons C.H., King A.J. (1999). Conductive hearing loss produces a reversible binaural hearing impairment. J. Neurosci..

[8] Schreiber B.E., Agrup C., Haskard D.O., Luxon L.M. (2010). Sudden sensorineural hearing loss. Lancet.

[9] Rosowski J.J., Cheng J.T., Merchant S.N., Harrington E., Furlong C. (2011). New data on the motion of the normal and reconstructed tympanic membrane. Otol. Neurotol. Off. Publ. Am. Otol. Soc. Am. Neurotol. Soc. Eur. Acad. Otol. Neurotol..

[10] Suzuki T., Ogiba Y. (1961). Conditioned orientation reflex audiometry: A new technique for pure-tone audiometry in young children under 3 years of age. Arch. Otolaryngol..

[11] Olsho L.W., Koch E.G., Carter E.A., Halpin C.F., Spetner N.B. (1988). Pure-tone sensitivity of human infants. J. Acoust. Soc. Am..

[12] Kujawa S.G., Liberman M.C. (2009). Adding insult to injury: Cochlear nerve degeneration after “temporary” noise-induced hearing loss. J. Neurosci..

[13] Whittemore K.R., Merchant S.N., Poon B.B., Rosowski J.J. (2004). A normative study of tympanic membrane motion in humans using a laser Doppler vibrometer (LDV). Hear. Res..

[14] Cheng J.T., Hamade M., Merchant S.N., Rosowski J.J., Harrington E., Furlong C. (2013). Wave motion on the surface of the human tympanic membrane: Holographic measurement and modeling analysis. J. Acoust. Soc. Am..

[15] Rosowski J.J., Nakajima H.H., Merchant S.N. (2008). Clinical utility of laser-Doppler vibrometer measurements in live normal and pathologic human ears. Ear Hear..

[16] Jakob A., Bornitz M., Kuhlisch E., Zahnert T. (2009). New aspects in the clinical diagnosis of otosclerosis using laser Doppler vibrometry. Otol. Neurotol..

[17] Foth H.-J., Huthoff C., Stasche N., Hoermann K. (1994). Measuring the motion of the human tympanic membrane by laser Doppler vibrometry: Basic principles and technical aspects. Proceedings of Microscopy, Holography, and Interferometry in Biomedicine.

[18] Wang X., Guan X., Pineda M., Gan R.Z. (2016). Motion of tympanic membrane in guinea pig otitis media model measured by scanning laser Doppler vibrometry. Hear. Res..

[19] Aarnisalo A.A., Cheng J.T., Ravicz M.E., Furlong C., Merchant S.N., Rosowski J.J. (2010). Motion of the tympanic membrane after cartilage tympanoplasty determined by stroboscopic holography. Hear. Res..

[20] De Greef D., Aernouts J., Aerts J., Cheng J.T., Horwitz R., Rosowski J.J., Dirckx J.J. (2014). Viscoelastic properties of the human tympanic membrane studied with stroboscopic holography and finite element modeling. Hear. Res..

[21] Khaleghi M., Furlong C., Ravicz M., Cheng J.T., Rosowski J. (2015). Three-dimensional vibrometry of the human eardrum with stroboscopic lensless digital holography. J. Biomed. Opt..

[22] Khaleghi M., Guignard J., Furlong C., Rosowski J.J. (2016). Full-Field Three-Dimensional Characterization of Non-repetitive Motions by Single-Shot Multiplexed Digital Holography. Experimental and Applied Mechanics, Volume 4.

[23] Cheng J.T., Aarnisalo A.A., Harrington E., del Socorro Hernandez-Montes M., Furlong C., Merchant S.N., Rosowski J.J. (2010). Motion of the surface of the human tympanic membrane measured with stroboscopic holography. Hear. Res..

[24] Huang D., Swanson E.A., Lin C.P., Schuman J.S., Stinson W.G., Chang W., Hee M.R., Flotte T., Gregory K., Puliafito C.A. (1991). Optical coherence tomography. Science.

[25] Fercher A.F., Drexler W., Hitzenberger C.K., Lasser T. (2003). Optical coherence tomography-principles and applications. Rep. Prog. Phys..

[26] Welzel J. (2001). Optical coherence tomography in dermatology: A review. Ski. Res. Technol. Rev. Artic..

[27] Lakshmikantha H.T., Ravichandran N.K., Jeon M., Kim J., Park H.-S. (2019). 3-Dimensional characterization of cortical bone microdamage following placement of orthodontic microimplants using Optical Coherence Tomography. Sci. Rep..

[28] Amaechi B.T., Higham S., Podoleanu A.G., Rogers J.A., Jackson D.A. (2001). Use of optical coherence tomography for assessment of dental caries: Quantitative procedure. J. Oral Rehabil..

[29] Meglinski I., Buranachai C., Terry L. (2010). Plant photonics: Application of optical coherence tomography to monitor defects and rots in onion. Laser Phys. Lett..

[30] Ravichandran N.K., Wijesinghe R.E., Shirazi M.F., Park K., Lee S.-Y., Jung H.-Y., Jeon M., Kim J. (2016). In vivo monitoring on growth and spread of gray leaf spot disease in capsicum annuum leaf using spectral domain optical coherence tomography. J. Spectrosc..

[31] Brown K., Harvey M. (2014). Optical coherence tomography: Age estimation of *Calliphora vicina* pupae in vivo?. Forensic Sci. Int..

[32] Ravichandran N., Wijesinghe R., Lee S.-Y., Choi K., Jeon M., Jung H.-Y., Kim J. (2017). Non-destructive analysis of the internal anatomical structures of mosquito specimens using optical coherence tomography. Sensors.

[33] Van der Jeught S., Dirckx J.J., Aerts J.R., Bradu A., Podoleanu A.G., Buytaert J.A. (2013). Full-field thickness distribution of human tympanic membrane obtained with optical coherence tomography. J. Assoc. Res. Otolaryngol..

[34] Nguyen C.T., Jung W., Kim J., Chaney E.J., Novak M., Stewart C.N., Boppart S.A. (2012). Noninvasive in vivo optical detection of biofilm in the human middle ear. Proc. Natl. Acad. Sci. USA.

[35] Chang E.W., Cheng J.T., Röösli C., Kobler J.B., Rosowski J.J., Yun S.H. (2013). Simultaneous 3D imaging of sound-induced motions of the tympanic membrane and middle ear ossicles. Hear. Res..

[36] Chen Z., Zhao Y., Srinivas S.M., Nelson J.S., Prakash N., Frostig R.D. (1999). Optical doppler tomography. IEEE J. Sel. Top. Quantum Electron..

[37] Yang V.X., Gordon M.L., Qi B., Pekar J., Lo S., Seng-Yue E., Mok A., Wilson B.C., Vitkin I.A. (2003). High speed, wide velocity dynamic range Doppler optical coherence tomography (Part I): System design, signal processing, and performance. Opt. Express.

[38] Yang V.X., Gordon M.L., Mok A., Zhao Y., Chen Z., Cobbold R.S., Wilson B.C., Vitkin I.A. (2002). Improved phase-resolved optical Doppler tomography using the Kasai velocity estimator and histogram segmentation. Opt. Commun..

[39] Izatt J.A., Kulkarni M.D., Yazdanfar S., Barton J.K., Welch A.J. (1997). In vivo bidirectional color Doppler flow imaging of picoliter blood volumes using optical coherence tomography. Opt. Lett..

[40] Chen Z., Milner T.E., Srinivas S., Wang X., Malekafzali A., van Gemert M.J., Nelson J.S. (1997). Noninvasive imaging of in vivo blood flow velocity using optical Doppler tomography. Opt. Lett..

[41] White B.R., Pierce M.C., Nassif N., Cense B., Park B.H., Tearney G.J., Bouma B.E., Chen T.C., de Boer J.F. (2003). In vivo dynamic human retinal blood flow imaging using ultra-high-speed spectral domain optical Doppler tomography. Opt. Express.

[42] Wang R.K., An L. (2009). Doppler optical micro-angiography for volumetric imaging of vascular perfusion in vivo. Opt. Express.

[43] Burkhardt A., Kirsten L., Bornitz M., Zahnert T., Koch E. (2014). Investigation of the human tympanic membrane oscillation ex vivo by Doppler optical coherence tomography. J. Biophotonics.

[44] Jeon D., Cho N.H., Park K., Kim K., Jeon M., Jang J.H., Kim J. (2019). In Vivo Vibration Measurement of Middle Ear Structure Using Doppler Optical Coherence Tomography: Preliminary Study. Clin. Exp. Otorhinolaryngol..

[45] Pawlowski M.E., Shrestha S., Park J., Applegate B.E., Oghalai J.S., Tkaczyk T.S. (2015). Miniature, minimally invasive, tunable endoscope for investigation of the middle ear. Biomed. Opt. Express.

[46] Kirsten L., Schindler M., Morgenstern J., Erkkilä M.T., Golde J., Walther J., Rottmann P., Kemper M., Bornitz M., Neudert M. (2018). Endoscopic optical coherence tomography with wide field-of-view for the morphological and functional assessment of the human tympanic membrane. J. Biomed. Opt..

[47] Park J., Cheng J.T., Ferguson D., Maguluri G., Chang E.W., Clancy C., Lee D.J., Iftimia N. (2016). Investigation of middle ear anatomy and function with combined video otoscopy-phase sensitive OCT. Biomed. Opt. Express.

[48] Ramier A., Cheng J.T., Ravicz M.E., Rosowski J.J., Yun S.-H. (2018). Mapping the phase and amplitude of ossicular chain motion using sound-synchronous optical coherence vibrography. Biomed. Opt. Express.

[49] MacDougall D., Farrell J., Brown J., Bance M., Adamson R. (2016). Long-range, wide-field swept-source optical coherence tomography with GPU accelerated digital lock-in Doppler vibrography for real-time, in vivo middle ear diagnostics. Biomed. Opt. Express.

[50] Kim W., Kim S., Huang S., Oghalai J.S., Applegate B.E. (2019). Picometer scale vibrometry in the human middle ear using a surgical microscope based optical coherence tomography and vibrometry system. Biomed. Opt. Express.

[51] Davis H., Hirsh S., Popelka G., Formby C. (1984). Frequency selectivity and thresholds of brief stimuli suitable for electric response audiometry. Audiology.

[52] Wijesinghe R.E., Park K., Kim D.-H., Jeon M., Kim J. (2016). In vivo imaging of melanoma-implanted magnetic nanoparticles using contrast-enhanced magneto-motive optical Doppler tomography. J. Biomed. Opt..

[53] Makita S., Fabritius T., Yasuno Y. (2008). Quantitative retinal-blood flow measurement with three-dimensional vessel geometry determination using ultrahigh-resolution Doppler optical coherence angiography. Opt. Lett..

[54] Ahn Y.-C., Jung W., Chen Z. (2007). Quantification of a three-dimensional velocity vector using spectral-domain Doppler optical coherence tomography. Opt. Lett..

[55] Zorowka P., Schmitt H., Gutjahr P. (1993). Evoked otoacoustic emissions and pure tone threshold audiometry in patients receiving cisplatinum therapy. Int. J. Pediatric Otorhinolaryngol..

[56] Selters W.A., Brackmann D.E. (1977). Acoustic tumor detection with brain stem electric response audiometry. Arch. Otolaryngol..

[57] Ravicz M.E., Tao Cheng J., Rosowski J.J. (2014). Sound pressure distribution within natural and artificial human ear canals: Forward stimulation. J. Acoust. Soc. Am..

[58] Araujo M.M., Massuda E.T., Hyppolito M.A. (2012). Anatomical and functional evaluation of tympanoplasty using a transitory natural latex biomembrane implant from the rubber tree *Hevea brasiliensis*. Acta Cir. Bras..

[59] Aernouts J., Soons J.A., Dirckx J.J. (2010). Quantification of tympanic membrane elasticity parameters from in situ point indentation measurements: Validation and preliminary study. Hear. Res..

