# Supplementary material for: In Situ Characterization of Micro-Vibration in Natural Latex Membrane Resembling Tympanic Membrane Functionally Using Optical Doppler Tomography
Source: Sensors (Basel). 2019 Dec 20;20(1):64. doi: 10.3390/s20010064 (PMC6982896; doi:10.3390/s20010064)
Supplement: Supplementary file 1 [file sensors-20-00064-s001.pdf]

sensors

MDPI

Supplementary Material

In Situ Characterization of Micro-Vibration in Natural Latex Membrane Resembling Tympanic Membrane Functionally Using Optical Doppler Tomography

Daewoon Seong [1,†], Jaehwan Kwon [1,†], Deokmin Jeon [1], Ruchire Eranga Wijesinghe [2], Jaeyul Lee [1], Naresh Kumar Ravichandran [1], Sangyeob Han [1], Junsoo Lee [1], Pilun Kim [3], Mansik Jeon [1,*] and Jeehyun Kim [1]

1 School of Electronics Engineering, College of IT engineering, Kyungpook National University, Daegu 41566, Republic of Korea; smc7095@knu.ac.kr (D.S.); luffy6462@naver.com (J.K.); dmjeon@knu.ac.kr (D.J.); jaeyul@knu.ac.kr (J.L.); nareshr.9169@gmail.com (N.K.R.); progkiller@paran.com (S.H.); jslee5399@knu.ac.kr (J.L.)

2 Department of Biomedical Engineering, College of Engineering, Kyungil University, Gyeongsan 38428, Republic of Korea; eranga@kiu.kr (R.E.W.)

3 School of Medicine, Institute of Biomedical Engineering, Kyungpook National University, Daegu 41944, Republic of Korea; pukim@knu.ac.kr (P.K.)

† These authors contributed equally to this work

* Correspondence: msjeon@knu.ac.kr; Tel.: +82-53-950-7846

Received: 30 October 2019; Accepted: 13 December 2019; Published: date

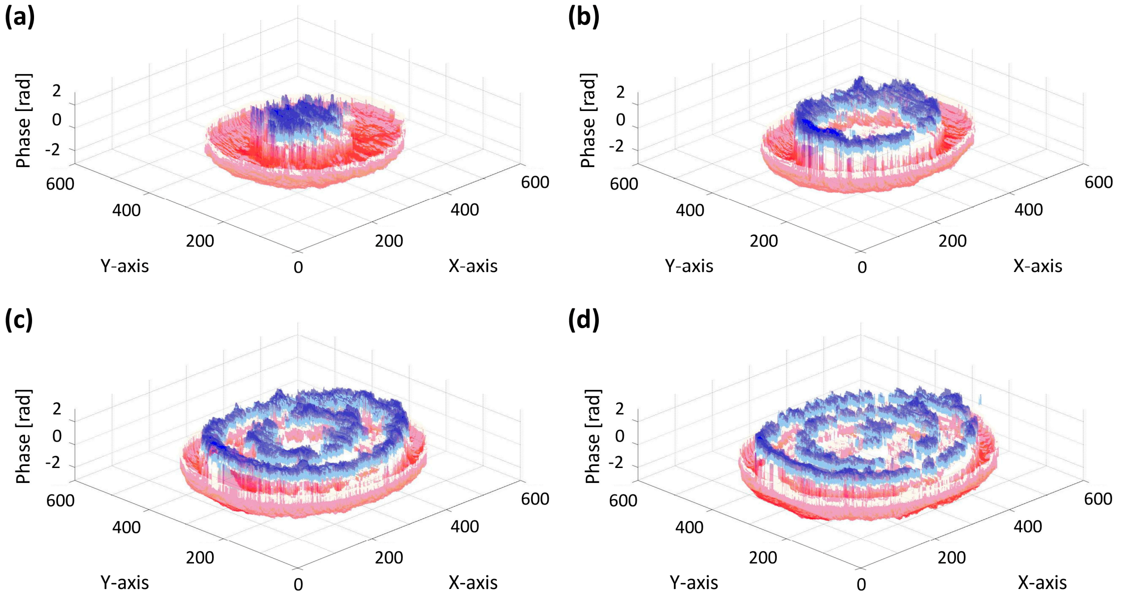

Figure 1. 3D-ODT mapping information to visualize direction of oscillation and correlated phase information for each scanning point. (**a**)–(**d**) depict the oscillation of latex membrane at 2.2 kHz, 2.8 kHz, 3.1 kHz and 3.2 kHz frequencies. Scale of x and y represent position of scanning and z is phase shift of vibration. Range of x and y axes are 0 to 600 identically because of the scanning points. Range of z axis is − 2 to +2 rad applied for Figure 4(**a**)–(**d**).

We implemented a 3D image in order to check the degree of phase shift due to the vibration of the latex membrane, which could not be seen in 2D at a glance. For 3D image processing, MATLAB software-based program was coded. The raw phase data obtained through the transverse scanning for

entire latex membrane were loaded into the program and each set of raw data was mapped according to the value set to draw the graph. Figure S1a–d are 3D images of the vibration of latex membrane when applied at 2.2 kHz, 2.8 kHz, 3.1 kHz and 3.2 kHz. The distinctive feature of each figure describes that the circular displacement structure is matched to a higher order of circular mode along with the frequency escalation. Figure S1a clearly shows that the latex membrane is oscillating in the second-order radial mode. Furthermore, mapping information at 2.8 kHz (Figure S1b) depicts (0, 3) radial mode, which is one step higher than the result at 2.2 kHz. Figure S1c,d show the modes of (0, 5) and (0, 6). Since the obtained results using latex membrane demonstrate micro-vibrations with respect to the frequency adjustments, a similarity between anatomical vibration patterns of TM and latex membrane was confirmed. Moreover, the anatomical structures, such as flattened and non-flattened regions of TM responds differently to frequency fluctuation, and therefore we applied different frequency levels to each different membrane location to gain a correlation with TM due to homogenous structural formation of latex membrane.

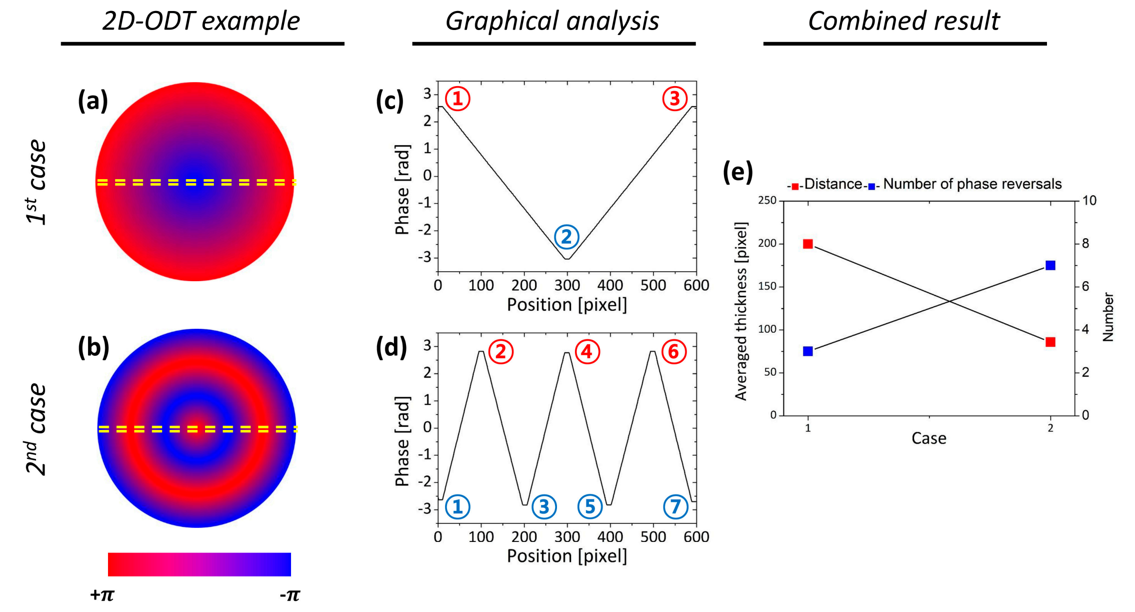

Figure 2. Entire process of plotting the Figure 4 using an example of 2D-ODT examples.

Figure S2 indicates the entire obtaining process of the Figure 5. We used 2 cases to explain the different result of different pattern as shown in below Figure S2a–b. Yellow-dashed lines indicate the averaging range, which contains 10 consecutive lines from the center (296 ~ 305 line). After averaging, we used a median filter and plotted the filtered data as shown in Figure S2c–d. The x-axis indicates the position of pixel and y-axis indicates phase information. In Figure S2c–d, we indicate the number of phase reversals using red and blue color numbers. Red number indicates the positive phase count and blue number is negative phase count. Figure S2c has total 3 phase reversals combined 2 positive and 1 negative counts. In addition, Figure S2d indicates 7 phase reversals with 3 positive and 4 negative counts. These values were shown in Figure S2e with blue color solid squares. For these two examples, total stimulated pixels were 600 and averaged pixels of rings were calculated as (600/number of phase reversals), which are shown in Figure S2e with red color solid squares. Our objective to calculate and compare the averaged thickness of rings is to verify the vibration complexity according to the applied frequency.
